# Obesity, birth weight, and lifestyle factors for frailty: a Mendelian randomization study

**DOI:** 10.18632/aging.205290

**Published:** 2023-12-12

**Authors:** Yingzhen Gu, Zuozhi Li, Aimin Dang, Wei Zhang, Jinxing Liu, Xiaorong Han, Yifan Li, Naqiang Lv

**Affiliations:** 1 Fuwai Hospital, National Center for Cardiovascular Diseases, Chinese Academy of Medical Science and Peking Union Medical College, Beijing, China

**Keywords:** obesity, lifestyle factors, frailty, Mendelian randomization

## Abstract

Obesity, birth weight and lifestyle factors have been found associated with the risk of frailty in observational studies, but whether these associations are causal is uncertain. We conducted a two-sample Mendelian randomization study to investigate the associations. Genetic instruments associated with the exposures at the genome-wide significance level (*p* < 5 × 10^−8^) were selected from corresponding genome-wide association studies (*n* = 143,677 to 703,901 individuals). Summary-level data for the frailty index were obtained from the UK Biobank (*n* = 164,610) and Swedish TwinGene (*n* = 10,616). The β of the frailty index was 0.15 (*p* = 3.88 × 10^−9^) for 1 standard deviation increase in the prevalence of smoking initiation, 0.19 (*p* = 3.54 × 10^−15^) for leisure screen time, 0.13 (*p* = 5.26 × 10^−7^) for body mass index and 0.13 (*p* = 1.80 × 10^−4^) for waist circumference. There was a suggestive association between genetically predicted higher birth weight and moderate-to-vigorous intensity physical activity with the decreased risk of the frailty index. We observed no causal association between genetically predicted age of smoking initiation and alcoholic drinks per week with the frailty index. This study supports the causal roles of smoking initiation, leisure screen time, overall obesity, and abdominal obesity in frailty. The possible association between higher birth weight, proper physical activity and a decreased risk of frailty needs further confirmation.

## INTRODUCTION

Frailty is characterized by a decline in physiological capacity across multiple systems, which leads to an increased vulnerability to stressors. It is highly prevalent in old age and is associated with a high risk of falls, disability, hospitalization, and mortality. This leads to a high burden of care and reduced quality of life [1–4]. As the aging population rapidly expands, the severity of frailty has gained increasing international attention. The Frailty index (FI), a relatively popular tool for measuring frailty, is a continuous measure calculated as the ratio of age-related health deficits to total deficits considered [5]. It has been previously validated in UK Biobank by Williams et al. [6]. It discriminates better at the low to middle end of the frailty continuum compared to the frailty phenotype, which is another measuring tool of frailty [7]. Several risk factors for frailty have been identified in previous studies, including smoking [8, 9], obesity [10–12], and sedentary behavior [13, 14], while alcohol [9, 15], birth weight [16], and physical activity [13, 14] have been identified as protective factors. However, most of the available evidence on frailty and these factors are from observational studies, which are vulnerable to potential confounders, reverse causality, and other biases that can undermine actual causation.

**Figure 1:**
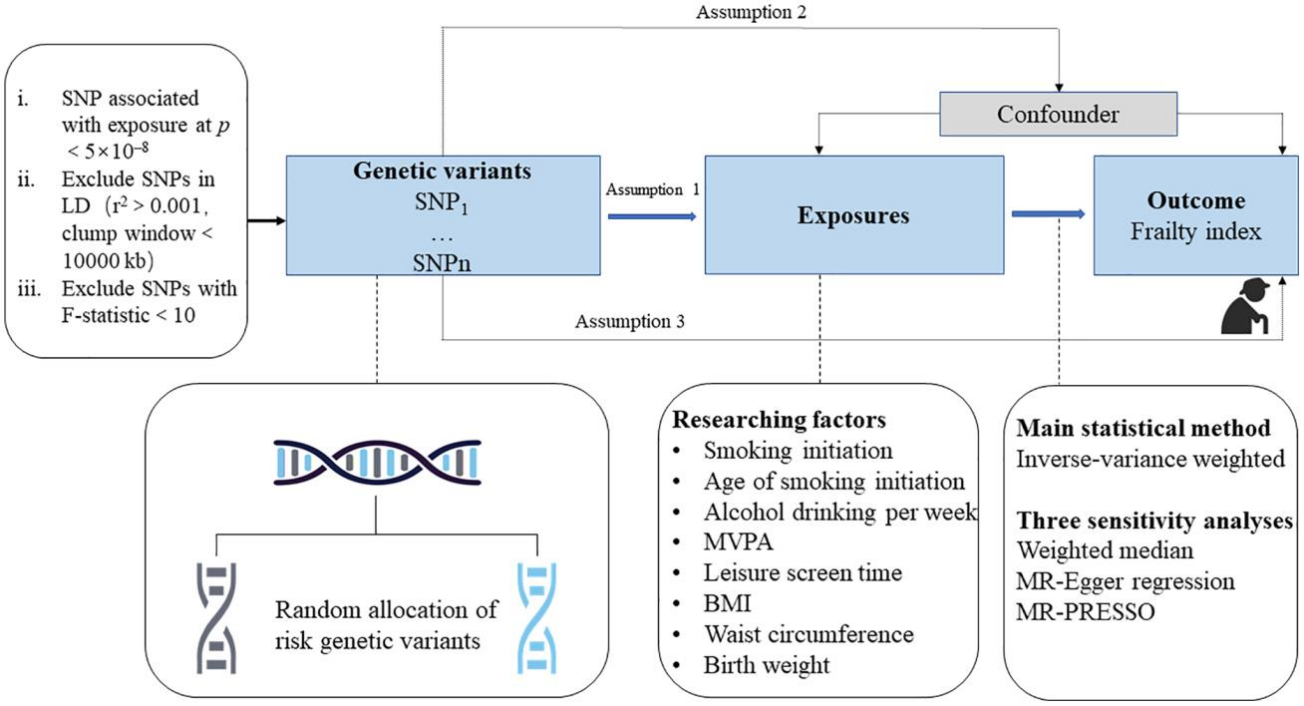
**Study design overview.** Abbreviations: SNP: single-nucleotide polymorphisms; LD: linkage disequilibrium; BMI: body mass index; MR-PRESSO: Mendelian randomization pleiotropy residual sum and outlier.

Mendelian randomization (MR) provides a novel analytic method that utilizes genetic variants of exposure as instrumental variables to estimate the causal association between exposure and health outcome [17]. Since genetic variants are randomly allocated and fixed at conception, MR studies can avoid reverse causality and are less vulnerable to confounders than conventional observational studies. Therefore, we applied two-sample MR to investigate the causal link between overall obesity (indicated by body mass index (BMI)), abdominal obesity (indicated by waist circumference), birth weight, and lifestyle factors (cigarette smoking, alcohol consumption, moderate-to-vigorous intensity physical activity (MVPA), and leisure screen time) and the risk of the frailty index (Figure 1).

## RESULTS

**Figure 2:**
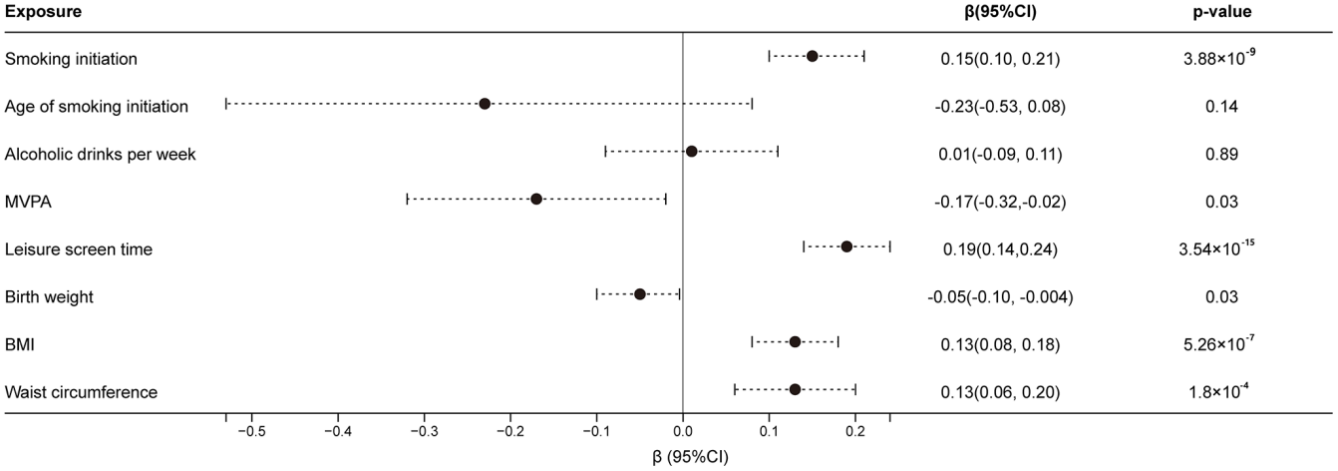
**Associations of genetically predicted factors with the risk of frailty index.** Estimates were obtained from the inverse-variance weighted method with random-effects. Abbreviations: CI: confidence interval; MVPA: moderate-to-vigorous intensity physical activity; BMI: body mass index.

According to the inverse-variance weighted (IVW) method, the principal statistical method, highly genetically predicted smoking initiation, longer leisure screen time, higher BMI and waist circumference were associated with an elevated risk of frailty (Figure 2). The β of the frailty index was 0.15 (95% confidence interval (CI), 0.10, 0.21; *p* = 3.88 × 10^−9^) for 1-standard deviation (SD) increase in the prevalence of smoking initiation, 0.19 (95% CI, 0.14, 0.24; *p* = 3.54 × 10^−15^) for 1-SD increase in leisure screen time, 0.13 (95% CI, 0.08, 0.18; *p* = 5.26 × 10^−7^) for 1-SD increase in BMI and 0.13 (95% CI, 0.06, 0.20; *p* = 1.80 × 10^−4^) for 1-SD increase in waist circumference. There was a suggestive association between genetically predicted higher birth weight (β for per 1-SD increase, −0.05, 95% CI, −0.10, −4.10 × 10^−3^; *p* = 0.03) and MVPA (β, −0.17, 95% CI, −0.32, −0.02; *p* = 0.03) with the risk of the frailty index. We observed no causal association between genetically predicted age of smoking initiation (β, −0.23, 95% CI, −0.53, 0.08; *p* = 0.14) and alcoholic drinks per week (β, 0.01, 95% CI, −0.09, 0.11; *p* = 0.89) with frailty index. In the three sensitivity analyses performed, the weighted median method and Mendelian randomization pleiotropy residual sum and outlier (MR-PRESSO) method remained completely consistent with the IVW method, with the MR-Egger regression method having a slightly different effect (Table 1).

**Table 1:** Association of genetically predicted risk factors with frailty index in MR sensitivity analysis.

**Risk factors**	**Cochrane’s Q**	***P*_pleiotropy_^a^**	***P*_distortion_^b^**	**MR-Egger**			**Weighted median**			**MR-PRESSO**			**Outliers**
				**β**	**95% CI**	***p***	**β**	**95% CI**	***p***	**β**	**95% CI**	***p***	
Smoking initiation	234.45	0.73	0.83	0.11	−0.15, 0.37	0.42	0.13	0.07, 0.18	4.79 × 10^−6^	0.14	0.09, 0.20	2.06 × 10^−7^	6
Age of smoking initiation	27.13	0.55	0.19	0.09	−0.92, 1.11	0.87	−0.08	−0.30, 0.14	0.47	−0.23	−0.53, 0.08	0.19	1
Alcoholic drinks per week	74.60	0.69	0.02	0.03	−0.14, 0.20	0.69	0.06	−0.04, 0.16	0.27	3.53 × 10^−3^	−0.10, 0.10	0.95	2
MVPA	17.76	0.97	0.92	−0.15	−0.87, 0.56	0.69	−0.17	−0.29, −0.04	8.26 × 10^−3^	−0.17	−0.26, −0.08	0.02	2
Leisure screen time	135.00	0.77	NA	0.16	−0.06, 0.38	0.15	0.15	0.09, 0.20	9.68 × 10^−8^	0.19	0.14, 0.24	4.96 × 10^−11^	0
Birth weight	71.05	0.96	0.20	−0.06	−0.22, 0.11	0.51	−0.04	−0.09, 0.02	0.21	−0.05	−0.10, −0.01	0.03	1
BMI	153.86	0.21	0.90	0.04	−0.11, 0.19	0.61	0.12	0.07, 0.17	6.54 × 10^−6^	0.12	0.07, 0.16	3.56 × 10^−6^	2
Waist circumference	111.35	0.80	0.35	0.10	−0.15, 0.35	0.43	0.12	0.05, 0.18	3.49 × 10^−4^	0.12	0.06, 0.19	4.06 × 10^−4^	4

Abbreviations: MVPA: moderate-to-vigorous intensity physical activity; BMI: body mass index; CI: confidence interval; NA: not available. *P*_pleiotropy_^a^ is the *p* values for MR-Egger intercept test and *p* value < 0.05 indicates a significant horizontal pleiotropy. *P*_distortion_^b^ is the *p* values obtained from MR-PRESSO distortion test and *p* value < 0.05 indicates a significant difference between estimates before and after outliers removal. *P*_distortion_ for leisure screen time was not available because of no outliers detected.

The F statistics for instrumental variants are shown in Table 2. They were all over 10, indicating the strong instrument strength of the single-nucleotide polymorphisms (SNPs) used. Horizontal pleiotropy of all the SNPs was not detected in the MR-Egger analysis (all of the *p*-values for intercept >0.05). Zero to six outliers were observed in MR-PRESSO analysis; however, the association of each exposure (except alcoholic drinks per week) with the frailty index remained consistent after the removal of the related outliers, and no difference was observed in estimates before and after removing outliers (*p* for distortion test >0.05) (Table 1).

**Table 2:** Detail information of corresponding studies.

**Exposure or outcome**	**Unit**	**Participants included in analysis**	**Adjustment**	**SNPs (selected instrumental variants)**	**F-statistic**	**PubMed ID**
Smoking initiation	SD in prevalence of smoking initiation	311,629 ever smokers and 321,173 never smokers of European-descent	Age, sex, and the first ten genetic principal components	93	30–145	30643251
Age of smoking initiation	SD	341,427 European-descent individuals	Age, sex, and the first ten genetic principal components	7	31–53	30643251
Alcoholic drinks per week	SD	335,394 European-descent individuals	Age, sex, and the first ten genetic principal components	35	30–927	30643251
MVPA	Dichotomous outcome (defined as at least 30 min per week of MVPA yes/no)	703,901 individuals (94.0% European, 2.1% African, 0.8% East Asian, 1.3% South Asian ancestries, and 1.9% Hispanic)	Age, sex and the first ten genetic principal components	9	31–91	36071172
Leisure screen time	SD	703,901 individuals (94.0% European, 2.1% African, 0.8% East Asian, 1.3% South Asian ancestries, and 1.9% Hispanic)	Age, sex and the first ten genetic principal components	89	27–111	36071172
Birth weight	SD	143,677 European-descent individuals	Gestational age	50	30–180	27680694
BMI	SD (>30 kg/m^2^)	322,154 European-descent individuals	Age, age squared, sex, and the first four genetic principal components	69	29–696	25673413
Waist circumference	SD	210,088 European-descent individuals	Age, age squared, and sex	42	29–447	25673412
Frailty index	SD	164,610 UK Biobank participants and 84,819 TwinGene participants of European descent	Age, sex, and the first ten genetic principal components	−	−	34431594

Abbreviations: SNPs: single-nucleotide polymorphisms; ID: identifier; SD: standard deviation; MVPA: moderate-to-vigorous intensity physical activity during leisure time; BMI: body mass index.

## DISCUSSION

This MR analysis revealed that smoking, longer leisure screen time, overall obesity, and abdominal obesity were causally associated with the risk of frailty. Additionally, it also suggested a possible causal link between higher birth weight, physical activity, and lower risk of frailty, but there was not enough evidence to support that alcohol consumption and age of smoking initiation were correlated with frailty.

Consistent with previous observations, this MR analysis indicated that smoking was associated with increased frailty regardless of the age at which smoking started. Similarly, the Rotterdam study with 11,539 participants reported that former and never smokers had lower FI scores than current smokers [18]. The Atlantic PATH cohort, comprising 9,133 participants aged 30–74 years, observed that current smokers of both genders under 60 years were more likely to have the highest level of frailty compared to never-smokers [9]. A possible mechanism for the smoking-related development of frailty could be smoking-induced DNA methylation [19].

Observational evidence on the association between alcohol consumption and frailty risk was not completely consistent. The Atlantic PATH cohort reported that female occasional alcohol drinkers were significantly less likely to be highly frail than non-drinkers, whereas, no significant association was found in the general population [9]. Another meta-analysis study suggested that heavier alcohol consumption was associated with lower incident frailty compared with no alcohol consumption [15]. This result was consistent with the result of the Rotterdam study showing that moderate or harmful alcohol intake was associated with less frailty than low alcohol intake [18]. This might be explained by the social benefits of drinking, such as enhancing positive situations and facilitating socializing with others, or by reversed causation, as those in poorer health were expected to stop their alcohol intake. Nevertheless, a study from Brazil did not find a significant association between alcohol consumption and frailty [20], which agreed with our MR results. Given the limited data and controversial results, more standardized studies are warranted.

Few studies are focusing on the effect of screen time on frailty. Nonetheless, enough evidence has proved that physical activity can help reduce frailty levels, while sedentary behaviors will accelerate the development of frailty [13, 21]. The underlined mechanism could be explained by the effects of physical exercise on anti-oxidative stress [22], anti-inflammation [23], and insulin resistance improvement [24]. As a type of sedentary behavior, longer screen time was significantly proved to be the obvious risk of frailty in our study, which verified the results of observational studies. However, the analysis of MR only pointed out that there was a suggestive association between MVPA and frailty index. This might be because the exposure used was a dichotomous variable, which hurt statistical power.

A growing body of evidence suggests a positive association between obesity and the risk of frailty. BMI, as a measure of overall obesity, also showed this association. A cohort study followed older adults (including 8,751 men and 3,033 women) for 26 years, finding that the risk of frailty increased with each additional year of obesity (BMI ≥30 kg/m^2^, adjusted OR 1.04 for men and 1.07 for women) [12]. Based on the 2001–2006 National Health and Nutrition Examination Survey (NHANES) cohorts and Survey of Health, Ageing and Retirement in Europe (SHARE), participants with a BMI level of ≥25 kg/m^2^ were found to have a higher level of frailty compared to those with a normal BMI [25]. Recent studies also obtained similar results using BMI ≥30 kg/m^2^ as a criterion [25, 26]. Waist circumference, as an indicator of abdominal obesity, is another appropriate way to evaluate obesity. In a meta-analysis of 12 observational studies comprising 37,985 older people, individuals with a BMI of ≥30 kg/m^2^ or a higher category of waist circumference were found to have a 40% or 57% higher risk of frailty, respectively, compared to those with normal values [10]. A cohort study also found that abdominal obesity was more closely associated with the incidence of frailty than overall obesity, and older adults with large waist circumferences are more likely to be frail [27]. Our data further confirmed this causal relationship. Several underlying mechanisms might explain the association between obesity and frailty. Obesity exacerbates the age-related decline in muscle mass, strength, cognitive impairment, and physical function [28–30], thus worsening health and well-being. It was also closely associated with metabolic disorders, inflammaging, insulin resistance, and oxidative stress [28, 31, 32], all of which have been proven risk factors for frailty [28, 31, 33, 34].

Previous review articles on the association between birth weight and frailty have found generally consistent evidence [16, 35, 36]. The Helsinki Birth cohort study, which included 1078 participants, observed that a 1 kg increase in birth weight was associated with a lower relative risk ratio (RRR) of frailty (RRR = 0.40), after adjusting for age and sex [16]. Recently, this cohort confirmed that for per 1 kg greater birth weight, the increase in FI levels per year was −0.087 percentage points slower [35]. Additionally, a longitudinal cohort study found that adults born with extremely low birth weight had reduced grip strength [36], which was a significant predictor of frailty. This finding is consistent with our MR results. Future well-designed studies are needed to explain the underlying mechanism.

As the progress of population aging accelerates, frailty is becoming a heavy burden in health and economy. From what we have found, it provided more sufficient evidence about different methods to decrease the risk of frailty. Reducing the number of smokers, shortening the leisure screen time and keeping a good shape will contribute to decrease the impact of frailty and improve living quality.

There are still several limitations that need to be considered when interpreting our results. The major issue for any MR analysis is the possible effect of horizontal pleiotropy, which means that the selected genetic instrumental variants influence the outcome not only via exposure but also via other potential confounders. However, in our analysis, it is unlikely that this limitation had a significant impact on our results. Firstly, all of the *p*-values for detecting pleiotropy from the MR-Egger intercept test had no statistical significance (*p* > 0.05). Secondly, the sensitivity analysis identified a few outliers by MR-PRESSO analysis, but the causal association remained consistent even after removing these outliers. Another limitation to consider is the partial overlap in the study population between the exposure and outcome, which may weaken the power of instrumental variants and bias causal estimates. Nevertheless, all the selected SNPs were at the high genome-wide significance level, and had F-statistic over 10, suggesting that the bias caused by partial overlap could be overlooked. Furthermore, our study was limited to individuals of European descent, which decreased the population structure bias. However, this confinement may limit the generalizability of our findings to other populations. Nevertheless, our findings were generally consistent with observational studies in patients of different descents, which strengthened the universality of the results. In our study, we used leisure screen time as a proxy for sedentary activity since the available genome-wide association studies (GWASs) data on sedentary activity mainly came from the UK Biobank, which had excessive overlaps with the outcome of the frailty index. We hope newer and more comprehensive GWAS studies will emerge to address this limitation in future studies.

## METHODS

### Study design and data sources

As shown in Figure 1, the study design overview should satisfy three assumptions: (i) the SNPs used as genetic instrumental variables should be strongly associated with exposure; (ii) the selected SNPs should not be correlated with potential confounders; and (iii) the SNPs used should affect the outcome only through the exposure, not through other alternative pathways [17]. All data in this study are based on the publicly available summary-level database from large GWASs and consortia.

### Genetic instrument selection

Genetic instrumental variants associated with smoking initiation [37], age of smoking initiation [37], alcoholic drinks per week [37], MVPA [38], leisure screen time [38], birth weight [39], BMI [40], and waist circumference [40] at the genome-wide significance level (*p* < 5 × 10^–8^) were obtained from corresponding GWASs (Table 2). The analysis of MVPA and leisure screen time was partially derived from multiple descents; therefore, we removed rs2173650, rs12981974, rs142601240 and rs9903845, retaining only the relative SNPs from European descents. We estimated the linkage disequilibrium (LD) among the SNPs by using the 1000 Genomes European panel as a reference population [41]. We excluded SNPs in LD (r^2^ > 0.001 and clump window < 10000 kb) and retained the SNPs with the strongest correlation to the exposure. The final SNP information is given in Supplementary Tables 1–8. We used F-statistics to evaluate the instrument strength of the relationship between each genetic instrumental variant and exposure. Generally, F <10 indicates weak instrument strength [42].

### Data sources for frailty index

Summary-level data on the associations of exposure-related SNPs with FI were obtained from a GWAS meta-analysis in European descent UK Biobank participants (*n* = 164,610, 60–70 years) and Swedish TwinGene participants (*n* = 10,616, 41–87 years) [43], which were available at Trait: Frailty index - IEU OpenGWAS project (mrcieu.ac.uk). FI was presented as the proportion of the sum of all deficits, which were based on 49 or 44 self-reported items on symptoms, disabilities, and diagnosed diseases for UK Biobank and TwinGene, respectively (see Supplementary Table 9 for details of the 49 items and the proportion of individuals scoring one for each item) [43]. The GWAS analysis was adjusted for age, sex, and the first ten principal components.

### Statistical analysis

We applied the random-effects IVW model as our primary statistical method, which gave the most precise estimate. However, the IVW model is susceptible to pleiotropy or invalid instrument bias if any of the assumptions are violated. To enhance the robustness of our results and test for pleiotropy, we performed three sensitivity analyses: the weighted median method, MR-Egger regression, and MR-PRESSO. The weighted median method can yield consistent estimates even when up to 50% of SNPs are invalid instrumental variables [44]. MR-Egger regression can adjust for pleiotropy but has low power. We used the *p* value of the MR-Egger intercept to detect horizontal pleiotropy [45]. The MR-PRESSO method can detect outliers and provide an estimate after the removal of outliers. The embedded distortion test is used to detect significant differences in the causal estimates before and after outlier removal [46]. Cochrane’s Q statistic was estimated to assess the heterogeneity of SNPs used for each exposure. To adjust for multiple testing, we applied a Bonferroni-corrected, two-sided significance level of 6.25 × 10^−3^ (0.05 divided by 8 risk exposures). We considered associations with *p*-value < 6.25 × 10^−3^ as significant, and associations with a *p*-value ≥ 6.25 × 10^−3^ and ≤ 0.05 as suggestive. We conducted all analyses using the TwoSampleMR package (version 0.5.6) in R (version 4.3.0).

## Supplementary Materials

Supplementary Tables
