# Supplementary material for: Obesity, birth weight, and lifestyle factors for frailty: a Mendelian randomization study
Source: Aging (Albany NY). 2023 Dec 12;15(23):14066–85. doi: 10.18632/aging.205290 (PMC10756094; doi:10.18632/aging.205290)
Supplement: Supplementary Tables [file aging-15-205290-s001.pdf]

SUPPLEMENTARY TABLES

Supplementary Table 1. The SNP information of smoking initiation.

SNP	Chr	Position	EA	OA	Beta	SE	*P*	EAF	*n*
rs3001723	1	44037685	A	G	0.033512	0.003898	8.12082E-18	0.321	632802
rs7555507	1	73766037	T	C	−0.02414	0.003556	1.13999E-11	0.496	632802
rs6669839	1	50625979	T	C	0.026004	0.004396	3.36001E-09	0.204	632802
rs12042107	1	91196176	C	T	−0.02228	0.003568	4.21998E-10	0.527	632802
rs2186122	1	66470206	T	A	0.026057	0.003586	3.60994E-13	0.561	632802
rs301805	1	8481016	G	T	0.021468	0.003613	2.80001E-09	0.559	632802
rs12025237	1	1.54E+08	C	A	−0.033	0.005339	6.52004E-10	0.124	632802
rs2050586	1	87905828	C	G	−0.02055	0.003708	2.99999E-08	0.355	632802
rs2046850	1	2.1E+08	T	C	−0.02481	0.004478	3.02998E-08	0.187	632802
rs6728726	2	623976	C	T	0.035449	0.004733	6.72977E-14	0.829	632802
rs78411160	2	58171220	C	A	0.020536	0.003659	2.03002E-08	0.631	632802
rs6433897	2	1.82E+08	C	T	0.022448	0.004058	3.16002E-08	0.754	632802
rs266047	2	1.04E+08	A	G	−0.03051	0.003739	3.3597E-16	0.529	632802
rs4674993	2	2.26E+08	G	A	−0.02521	0.004436	1.31999E-08	0.207	632802
rs578584	2	45143175	T	A	0.02868	0.003596	1.50003E-15	0.605	632802
rs35702515	2	1.38E+08	T	G	0.025244	0.004231	2.43002E-09	0.162	632802
rs13030994	2	1.46E+08	A	G	0.036093	0.003556	3.56041E-24	0.485	632802
rs12474587	2	1.63E+08	T	G	0.027633	0.003582	1.24997E-14	0.404	632802
rs2107300	2	2.01E+08	G	C	−0.0272	0.004925	3.27002E-08	0.845	632802
rs7585579	2	60024857	G	C	0.0224	0.003728	1.88001E-09	0.505	632802
rs1445649	2	1.56E+08	C	T	0.023993	0.003565	1.67996E-11	0.525	632802
rs6788098	3	85624131	T	A	−0.03135	0.003689	1.90985E-17	0.623	632802
rs12632110	3	50224225	G	A	−0.02338	0.003753	4.78002E-10	0.647	632802
rs11712680	3	75009019	C	A	−0.02705	0.004578	3.51002E-09	0.174	632802
rs1154693	3	1.18E+08	G	A	0.032622	0.004912	3.12033E-11	0.856	632802
rs66680800	3	85985324	T	G	−0.02027	0.003653	2.83002E-08	0.397	632802
rs1869243	3	5724536	C	T	0.019741	0.003563	2.97002E-08	0.481	632802
rs9835772	3	85766025	T	A	0.024047	0.004142	6.32004E-09	0.235	632802
rs962625	4	28473524	G	A	0.023718	0.004038	4.36999E-09	0.24	632802
rs993700	4	67825894	C	T	−0.02593	0.004292	1.52999E-09	0.766	632802
rs13145728	4	1.41E+08	C	G	−0.02325	0.003663	2.13998E-10	0.358	632802
rs10001365	4	1.48E+08	A	G	−0.02499	0.003642	6.64967E-12	0.405	632802
rs1160685	4	94052854	G	C	0.020772	0.003589	7.19996E-09	0.478	632802
rs6893752	5	60374912	G	A	−0.0241	0.004074	3.24997E-09	0.766	632802
rs12186738	5	1.04E+08	T	G	−0.03326	0.005021	3.41979E-11	0.154	632802
rs1385108	5	1.55E+08	T	C	0.024662	0.004157	2.99999E-09	0.239	632802
rs4044321	5	1.67E+08	G	A	−0.02784	0.003711	6.07995E-14	0.642	632802
rs4352629	5	87756821	T	C	−0.02753	0.003569	1.22011E-14	0.492	632802
rs72789632	5	1.07E+08	T	C	−0.03289	0.005286	5.01996E-10	0.12	632802
rs9401770	6	98748008	A	G	0.027731	0.003986	3.46976E-12	0.273	632802
rs222449	6	52916062	T	A	−0.02532	0.004428	1.07999E-08	0.793	632802
rs3800227	6	1.09E+08	G	A	0.022812	0.004058	1.93001E-08	0.701	632802
rs10498846	6	67405337	T	C	0.02061	0.003556	6.62003E-09	0.473	632802
rs240963	6	1.12E+08	C	T	−0.04104	0.004837	2.16023E-17	0.836	632802
rs12333760	7	99185406	C	T	−0.02905	0.004801	1.43999E-09	0.204	632802
rs10233018	7	1.18E+08	G	A	0.027069	0.003557	2.74979E-14	0.503	632802
rs10279261	7	1.34E+08	A	G	−0.02142	0.003663	0.000000005	0.619	632802
rs10260968	7	1889773	A	G	−0.02032	0.003609	1.75001E-08	0.597	632802
rs12112638	7	69735251	G	A	−0.02453	0.004043	1.33999E-09	0.275	632802
rs4236259	7	1708080	G	T	−0.02477	0.003557	3.34965E-12	0.499	632802
rs2140114	7	3407568	T	C	−0.02326	0.003734	4.70002E-10	0.518	632802
rs3801289	7	96638267	C	A	−0.02206	0.00374	3.73999E-09	0.351	632802

rs1565735	8	27426077	A	T	−0.03762	0.004461	3.41979E-17	0.212	632802
rs1899896	8	93201036	T	C	0.026448	0.003887	1.03992E-11	0.286	632802
rs13261666	8	59814666	T	G	−0.02689	0.003556	3.90032E-14	0.522	632802
rs12545053	8	65073605	G	A	0.020281	0.003637	2.43002E-08	0.397	632802
rs2631024	8	91995577	G	A	−0.02296	0.004028	1.17999E-08	0.737	632802
rs4543592	9	3014254	C	T	0.021931	0.003562	7.46002E-10	0.468	632802
rs2378662	9	86707289	A	G	0.020948	0.003566	4.15997E-09	0.556	632802
rs10114490	9	11070165	A	G	−0.02551	0.004532	1.81001E-08	0.198	632802
rs10905461	10	8803551	C	T	−0.02396	0.004145	7.35005E-09	0.718	632802
rs7921378	10	63674885	C	G	−0.02546	0.003558	8.26038E-13	0.463	632802
rs12356821	10	1.05E+08	C	G	0.03937	0.005049	6.27047E-15	0.14	632802
rs10159545	10	21766969	G	C	0.02625	0.003727	1.83992E-12	0.375	632802
rs9423279	10	1.26E+08	G	C	−0.02051	0.003708	3.21004E-08	0.641	632802
rs7938812	11	1.13E+08	G	T	0.043791	0.003637	2.71019E-33	0.424	632802
rs6265	11	27679916	T	C	−0.03179	0.004578	3.76964E-12	0.203	632802
rs7929518	11	85980958	G	A	0.024238	0.004285	1.55998E-08	0.765	632802
rs4523689	11	7950797	G	A	−0.02061	0.003643	1.54999E-08	0.408	632802
rs11057005	12	16748721	G	A	−0.02093	0.003579	4.84998E-09	0.43	632802
rs4759228	12	56508409	C	G	−0.02169	0.003934	3.57998E-08	0.27	632802
rs7969559	12	69655167	G	A	−0.02438	0.003959	7.31004E-10	0.688	632802
rs1971318	12	1.21E+08	T	C	0.028507	0.004925	7.05992E-09	0.141	632802
rs7322872	13	1.01E+08	T	C	−0.02557	0.004335	3.57998E-09	0.782	632802
rs3904512	13	38357471	A	G	−0.02116	0.003577	3.22998E-09	0.429	632802
rs9540729	13	66947124	T	A	−0.01955	0.003558	3.81997E-08	0.501	632802
rs76214862	14	29500130	C	A	−0.02499	0.004547	3.98997E-08	0.202	632802
rs12441907	15	83922387	A	C	−0.02921	0.004523	1.06001E-10	0.186	632802
rs1435741	15	47935843	A	G	0.029415	0.003591	2.63998E-16	0.425	632802
rs4785836	16	65604652	C	T	−0.02047	0.003659	2.26001E-08	0.398	632802
rs7197072	16	717085	T	C	−0.02477	0.004169	2.77E-09	0.238	632802
rs1050847	16	87443734	T	C	−0.02162	0.003589	1.67001E-09	0.505	632802
rs4781977	16	17572674	C	T	−0.02387	0.004365	4.54004E-08	0.205	632802
rs11078713	17	7795972	G	A	−0.02017	0.003606	2.22998E-08	0.454	632802
rs7224742	17	30657058	T	C	−0.02071	0.003655	1.43001E-08	0.595	632802
rs11658881	17	2072949	G	A	0.020136	0.003611	2.43002E-08	0.418	632802
rs6508144	18	50026142	G	C	−0.02069	0.003586	7.97003E-09	0.563	632802
rs11872397	18	72535282	A	G	−0.02477	0.004095	1.43001E-09	0.252	632802
rs72896886	18	42632652	C	G	−0.02689	0.004837	2.74998E-08	0.144	632802
rs76608582	19	4474725	A	C	−0.04956	0.00826	1.93999E-09	0.0389	632802
rs1555445	20	31175258	T	A	0.022555	0.003823	3.64998E-09	0.337	632802
rs117143374	21	40555561	C	T	0.02929	0.005269	2.76001E-08	0.12	632802
rs134529	22	28781758	C	T	−0.01998	0.003661	4.84998E-08	0.349	632802

Abbreviations: EA: effect allele; OA: other allele; EAF: effect allele frequency; *n*: sample size; beta: effect size estimate: SE: standard error; *P*: *p*-value.

Supplementary Table 2. The SNP information of age of smoking initiation.

SNP	Chr	Position	EA	OA	Beta	SE	*P*	EAF	*n*
rs10200107	2	63613544	A	G	−0.01956	0.002777	1.83992E-12	0.558	257349
rs3768886	2	225450161	C	G	0.01714	0.002966	7.37004E-09	0.328	258495
rs11915747	3	85699040	G	C	0.02099	0.002891	3.83001E-13	0.354	258249
rs624833	4	2881256	G	T	0.017295	0.003006	8.60994E-09	0.309	260302
rs11780471	8	27344719	A	G	0.03785	0.005806	7.00003E-11	0.0644	252818
rs140485736	15	75360268	A	G	0.065453	0.011535	1.41E-08	0.0131	221509
rs319748	17	31554533	A	G	−0.01703	0.003074	3.07999E-08	0.711	260875

Abbreviations: EA: effect allele; OA: other allele; EAF: effect allele frequency; *n*: sample size; beta: effect size estimate: SE: standard error; *P*: *p*-value.

Supplementary Table 3. The SNP information of alcoholic drinks per week.

SNP	Chr	Position	EA	OA	Beta	SE	*P*	EAF	*n*
rs28680958	1	1.74E+08	A	G	−0.01358	0.002368	9.78003E-09	0.23	532042
rs1260326	2	27730940	C	T	0.023812	0.001984	3.32966E-33	0.595	532340
rs6739804	2	63269604	C	T	−0.01297	0.002082	4.71998E-10	0.66	532202
rs75120545	2	44271496	T	C	−0.03279	0.005673	7.58997E-09	0.022	474779
rs71404478	2	1.44E+08	T	C	−0.01259	0.002011	3.83001E-10	0.34	511751
rs494904	2	45141180	C	T	0.015085	0.001961	1.40994E-14	0.429	531073
rs9835772	3	85766025	T	A	0.013204	0.002242	3.89996E-09	0.235	530478
rs28732378	3	85403892	G	A	−0.01673	0.002191	2.23975E-14	0.729	530009
rs16854020	4	42117559	A	G	0.018083	0.002907	4.82003E-10	0.127	533501
rs78234152	4	1E+08	A	G	0.027654	0.003071	2.18022E-19	0.0986	534076
rs331939	4	1.44E+08	A	G	−0.0119	0.002029	4.49997E-09	0.339	536700
rs1229984	4	1E+08	C	T	0.188115	0.006179	1E-200	0.953	514602
rs13107325	4	1.03E+08	T	C	−0.03645	0.003913	1.22999E-20	0.0654	528164
rs28712821	4	39413780	A	G	0.028334	0.001974	1.10002E-46	0.594	525616
rs55872084	5	1.56E+08	T	G	0.012731	0.002268	1.98002E-08	0.218	529669
rs6969458	7	1.53E+08	A	G	0.012706	0.001935	5.19996E-11	0.459	509646
rs2299409	7	1.04E+08	A	G	−0.01055	0.001933	4.79999E-08	0.493	534249
rs10085696	7	69783020	G	A	−0.01605	0.002495	1.24E-10	0.201	535056
rs28601761	8	1.27E+08	G	C	0.011299	0.001955	7.59994E-09	0.405	523035
rs55932213	9	1.09E+08	G	A	0.012477	0.002216	1.79999E-08	0.701	515956
rs4309187	11	1.13E+08	C	A	0.014791	0.002088	1.36994E-12	0.697	531063
rs17542254	11	1.14E+08	G	A	0.013142	0.002146	8.96004E-10	0.251	533884
rs2049045	11	27694241	C	G	−0.01377	0.002505	3.97E-08	0.189	532726
rs4752999	11	47428565	T	C	−0.01456	0.00207	2.03002E-12	0.321	529379
rs1387766	12	92081800	A	G	−0.01083	0.001983	4.79005E-08	0.622	535067
rs28929474	14	94844947	T	C	−0.04768	0.00714	2.39001E-11	0.0154	528430
rs962961	14	57281154	T	C	−0.01219	0.002051	2.78003E-09	0.329	533853
rs11860773	16	73912503	C	T	−0.01501	0.002444	8.35007E-10	0.176	515422
rs153106	16	28526897	C	T	−0.01361	0.001959	3.62994E-12	0.409	531637
rs13332432	16	85721809	G	C	0.014005	0.002141	5.94019E-11	0.296	516946
rs79616692	16	72338507	C	G	0.018809	0.003152	2.38002E-09	0.11	529527
rs76640332	17	44189858	A	G	−0.02101	0.002389	1.46994E-18	0.204	514949
rs34121753	17	7733833	G	A	0.011069	0.001951	1.38998E-08	0.532	518183
rs676388	19	49211969	C	T	0.015098	0.001931	5.49035E-15	0.494	531996
rs6106989	20	25027630	A	G	0.010899	0.001983	3.80996E-08	0.628	520623

Abbreviations: EA: effect allele; OA: other allele; EAF: effect allele frequency; *n*: sample size; beta: effect size estimate: SE: standard error; *P*: *p*-value.

Supplementary Table 4. The SNP information of moderate-to-vigorous intensity physical activity during leisure time.

SNP	Chr	Position	EA	OA	Beta	SE	P	EAF	n
rs1160545	2	100832269	T	C	0.0249	0.0041	1.73E-09	0.4025	483768
rs7613360	3	49916710	C	T	0.0247	0.0042	2.77E-09	0.6037	483768
rs1691471	3	85011013	T	C	0.0379	0.0042	1.73E-19	0.3759	483768
rs182484063	4	28821739	C	A	0.13	0.021	2.09E-09	0.02	219777
rs150882750 (aka rs10673865)	4	109098823	T	TAC	0.04	0.006	3.17E-09	0.54	243769
rs13201721	6	141799534	T	C	0.0255	0.004	1.83E-10	0.7364	522597
rs1625595	11	66078129	C	T	0.0213	0.0032	1.9E-11	0.5252	592552
rs7946119	11	16421858	C	T	0.04	0.006	5.37E-11	0.4	243411
rs385301	17	19806828	C	T	0.0284	0.0047	1.6E-09	0.7363	461744

Abbreviations: EA: effect allele; OA: other allele; EAF: effect allele frequency; *n*: sample size; beta: effect size estimate: SE: standard error; *P*: *p*-value.

Supplementary Table 5. The SNP information of leisure screen time.

SNP	Chr	Position	EA	OA	Beta	SE	*P*	EAF	*n*
rs3791033	1	44134077	C	T	−0.033	0.004	3.66E-16	0.331	469809
rs144839021	1	98342685	C	T	−0.296	0.044	2.41E-11	0.998	526370
rs197439	1	112280990	A	G	−0.026	0.004	3.29E-11	0.601	469809
rs71658797	1	77967507	T	A	−0.037	0.006	1.81E-10	0.878	469809
rs10889193	1	61106174	C	A	−0.024	0.004	4.94E-10	0.445	469809
rs6685030	1	171805284	A	G	−0.022	0.004	5.27E-10	0.48	526371
rs543154297 (akars34770465)	1	243921068	CTTT	C	−0.031	0.005	2.94E-09	0.184	442658
rs10189857	2	60713235	A	G	−0.027	0.004	7.8E-15	0.565	525491
rs144155998	2	147890505	TA	T	−0.043	0.006	1.3E-14	0.85	442658
rs4303732	2	100830040	C	T	−0.027	0.004	5.37E-14	0.402	525479
rs12617870	2	193746283	G	T	−0.026	0.004	6.62E-14	0.462	525491
rs1160545	2	100832269	T	C	−0.029	0.004	1.14E-13	0.403	468924
rs114590429	2	166176789	C	A	−0.038	0.006	3.03E-10	0.884	468924
rs36079846	2	215367159	T	C	−0.024	0.004	4.52E-10	0.521	468924
rs566017137	2	157149797	C	G	−0.066	0.011	1.23E-09	0.964	442658
rs145255225 (akars34908368)	2	44255167	CAT	C	−0.025	0.004	1.28E-09	0.575	442658
rs12992995	2	175197545	A	C	−0.026	0.004	2.1E-09	0.276	468924
rs62131183	2	45102896	A	G	−0.054	0.009	3.31E-09	0.047	468830
rs62151809	2	104433256	C	T	−0.023	0.004	3.9E-09	0.547	468924
rs7615206	3	49937505	T	C	−0.035	0.004	1.5E-22	0.57	521894
rs7613360	3	49916710	C	T	−0.032	0.004	6.92E-16	0.604	468924
rs9867121	3	114631548	A	C	−0.032	0.005	2.02E-10	0.183	468924
rs1375561	3	85658230	C	T	−0.023	0.004	2.55E-10	0.347	525491
rs76267866	3	70540347	A	T	−0.03	0.005	3.53E-10	0.792	468924
rs150123913	3	165781804	T	TA	−0.025	0.004	8.92E-10	0.605	442658
rs62244886	3	71587392	G	C	−0.024	0.004	1.24E-09	0.396	468924
rs7430216	3	75201030	C	T	−0.025	0.004	2.5E-09	0.777	523040
rs7432837	3	93809151	T	C	−0.024	0.004	3.24E-09	0.742	525491
rs9821299	3	83068067	G	A	−0.029	0.005	3.63E-09	0.815	482490
rs17025214	3	88023541	T	C	−0.022	0.004	4.31E-09	0.31	525487
rs548236486 (akars199551309)	4	17922813	A	AT	−0.038	0.006	1.45E-10	0.868	442658
rs743699	4	3305116	A	G	−0.027	0.004	1.18E-09	0.743	468924
rs4416502	4	77030872	G	A	−0.029	0.005	1.38E-09	0.8	468924
rs13107325	4	103188709	C	T	−0.04	0.007	1.79E-09	0.926	525490
rs262890	5	62930015	A	G	−0.034	0.004	2.06E-16	0.7	468924
rs752485316	5	138355612	CT	C	−0.032	0.004	3.1E-14	0.669	442658
rs67777640	5	77489568	T	TTATA	−0.029	0.004	8.44E-12	0.654	442658
rs1947066	5	161101615	G	A	−0.03	0.004	8.54E-12	0.197	525491
rs2964252	5	152067929	A	G	−0.024	0.004	3.16E-10	0.316	525491
rs396321	5	112113735	T	C	−0.021	0.004	1.29E-09	0.513	521893
rs249960	5	96164771	G	A	−0.03	0.005	2.43E-09	0.182	468924
rs200307517	6	139259142	C	CT	−0.028	0.004	1.45E-10	0.352	442658
rs58541850	6	166165563	G	A	−0.052	0.008	1.72E-10	0.942	468924
rs558134	6	12693454	T	C	−0.023	0.004	5.05E-10	0.384	525479
rs12324720	6	107649123	A	G	−0.027	0.005	3.53E-09	0.176	468924
rs6457816	6	35362848	T	C	−0.041	0.007	3.97E-09	0.934	525488
rs543294537 (akars11419307)	7	99118077	AT	A	−0.04	0.006	6.39E-13	0.154	442658
rs12062845	7	101870885	C	A	−0.028	0.004	2.41E-11	0.784	442658
rs13235840	7	133505091	A	T	−0.031	0.005	3.48E-10	0.819	468924
rs531112651 (akars200756759)	7	53853183	G	GA	−0.03	0.005	6.95E-10	0.783	442658
rs2529484	7	111180444	G	C	−0.022	0.004	1.39E-09	0.649	525488

rs17621391	7	140176596	C	T	−0.024	0.004	2.11E-09	0.265	525485
rs55751618	8	30862954	CA	C	−0.033	0.004	8.3E-15	0.631	442658
rs12678836	8	92690148	C	A	−0.023	0.004	5.37E-11	0.576	524165
rs7821826	8	10769439	C	T	−0.021	0.004	3.25E-09	0.508	524166
rs1999065	9	120514574	C	T	−0.025	0.004	1.24E-11	0.662	526725
rs73581580	9	140251458	G	A	−0.037	0.006	1.94E-10	0.876	468924
rs2783992	9	1722044	T	C	−0.024	0.004	3.36E-10	0.539	468924
rs34864022	9	22609110	A	G	−0.048	0.008	4.71E-10	0.934	468924
rs566556664	9	134723482	GTGTGGT GTGTGCA	G	−0.036	0.006	3.22E-09	0.866	442658
rs11275375	10	21839820	T	TAATTAAA AATTATGT GAAAAC	−0.042	0.004	2.59E-22	0.678	442658
rs68049022	10	66407019	C	T	−0.031	0.005	6.18E-11	0.202	468924
rs841020	10	125409953	C	T	−0.027	0.004	1.15E-09	0.808	525491
rs4483592	11	65990439	C	T	−0.036	0.005	3.97E-12	0.837	468924
rs4551799	11	71416091	A	G	−0.024	0.004	6.91E-10	0.344	512111
rs1391954	11	88575965	G	T	−0.025	0.004	1.51E-09	0.556	442658
rs3759344	12	6862646	G	A	−0.045	0.006	4.26E-13	0.894	468924
rs7969719	12	109883577	C	T	−0.027	0.004	4.46E-13	0.687	525490
rs541140319 (akars59131741)	12	123536456	C	CT	−0.028	0.005	4.11E-10	0.314	442658
rs10772643	12	13415288	T	C	−0.039	0.006	5.88E-10	0.892	468924
rs142471757	12	24102749	CCT	C	−0.028	0.005	1.65E-09	0.735	442658
13:100753041ctc	13	100753041	CT	C	−0.036	0.004	3.17E-17	0.634	442658
rs9513416	13	99055774	G	A	−0.028	0.005	4.1E-09	0.159	525490
rs10400776	14	97326366	A	C	−0.026	0.004	3.45E-09	0.259	468924
rs56151256	15	78024806	C	A	−0.029	0.004	1.17E-10	0.25	468924
rs78394231	15	64092140	T	C	−0.038	0.007	3.53E-09	0.902	525490
rs4889530	16	31065918	A	T	−0.025	0.004	1.32E-10	0.381	468924
rs11074658	16	10308335	T	C	−0.024	0.004	9.21E-10	0.591	468924
rs1860337	17	60851559	T	C	−0.025	0.004	9.08E-11	0.595	468924
rs73420302	17	77768068	C	G	−0.03	0.005	3.04E-09	0.179	468924
rs148544378	18	40323567	T	C	−0.088	0.014	6.55E-11	0.021	468924
rs12962050	18	35179808	A	G	−0.023	0.004	1.18E-10	0.647	525490
rs78140587	18	41817809	G	A	−0.062	0.01	5.83E-10	0.041	468924
rs6857	19	45392254	T	C	−0.037	0.005	5.8E-15	0.169	523876
rs2229383	19	10794630	T	G	−0.029	0.004	6.4E-13	0.635	468924
rs139900206 (akars3838037)	20	43525483	T	TTA	−0.042	0.006	9.36E-12	0.871	442658
rs6010651	20	62418243	C	A	−0.024	0.004	3.34E-09	0.38	468924
rs7067170	23	68382836	G	A	−0.026	0.004	8.66E-11	0.762	442658
rs1604558	23	39198051	A	G	−0.023	0.004	1.36E-09	0.74	442658

Abbreviations: EA: effect allele; OA: other allele; EAF: effect allele frequency; *n*: sample size; beta: effect size estimate: SE: standard error; *P*: *p*-value.

Supplementary Table 6. The SNP information of birth weight.

SNP	Chr	Position	EA	OA	Beta	SE	*P*	EAF	*n*
rs3753639	1	1.55E+08	C	T	0.0306	0.0045	7.29962E-12	0.24	138162
rs72480273	1	1.62E+08	C	A	0.0313	0.0051	8E-10	0.17	138380
rs2473248	1	22536643	C	T	0.0325	0.0057	0.00000001	0.87	139428
rs17034876	2	46484310	T	C	0.0471	0.0042	2.60016E-29	0.7	134460
rs7575873	2	23962647	G	A	−0.0384	0.0057	1.20005E-11	0.12	139425
rs10935733	3	1.49E+08	C	T	−0.0221	0.0039	9.20005E-09	0.59	139426
rs11720108	3	1.23E+08	T	C	0.046	0.0043	3.40017E-26	0.23	143673
rs2168443	3	46947087	A	T	−0.0228	0.0039	3.50002E-09	0.62	139426
rs900399	3	1.57E+08	G	A	−0.0523	0.0039	2.19989E-41	0.39	143663

rs2131354	4	1.46E+08	A	G	0.0259	0.0037	4.10015E-12	0.53	139431
rs4144829	4	17903654	T	C	−0.0341	0.0042	5.30029E-16	0.73	139426
rs854037	5	57091783	G	A	−0.0268	0.0048	2.19999E-08	0.19	139429
rs2946179	5	1.58E+08	C	T	0.024	0.0042	1.29999E-08	0.73	143666
rs1415701	6	1.3E+08	A	G	−0.0253	0.0043	2.59998E-09	0.26	143666
rs35261542	6	20675792	A	C	−0.0444	0.0041	4.40048E-27	0.27	143667
rs9368777	6	33788637	C	G	0.0215	0.0038	2.19999E-08	0.58	135709
rs1187118	6	34169020	T	A	−0.0299	0.0051	3.59998E-09	0.83	137043
rs10872678	6	1.52E+08	C	T	−0.0375	0.0041	6.90081E-20	0.28	143672
rs138715366	7	44246271	T	C	−0.2412	0.0229	7.19946E-26	0.0089	132343
rs111778406	7	72957570	G	A	0.0492	0.0075	5.79963E-11	0.068	140932
rs798498	7	2795882	G	T	−0.0229	0.004	1.29999E-08	0.31	139427
rs11765649	7	23479013	C	T	−0.0267	0.0043	5.80003E-10	0.25	139428
rs12543725	8	1.42E+08	A	G	−0.0231	0.0038	1.2E-09	0.41	139431
rs13266210	8	41533514	G	A	−0.0308	0.0045	1.29987E-11	0.21	139429
rs7854962	9	96900505	G	C	−0.0279	0.0046	1.89998E-09	0.22	139424
rs1411424	9	1.14E+08	A	G	0.0212	0.0038	2.19999E-08	0.52	139428
rs3780573	9	98239503	A	G	0.0555	0.0064	7.00003E-18	0.096	134750
rs10818797	9	1.26E+08	C	T	0.0345	0.0054	1.2E-10	0.14	139427
rs79237883	10	1.05E+08	C	T	0.0371	0.0067	3.50002E-08	0.08	143666
rs2497304	10	94492716	T	C	−0.0282	0.0037	2.60016E-14	0.48	143673
rs740746	10	1.16E+08	A	G	0.0364	0.0042	3.80014E-18	0.73	143672
rs10830963	11	92708710	G	C	0.0232	0.0042	2.90001E-08	0.28	143663
rs72851023	11	2130620	T	C	0.0476	0.0075	2.90001E-10	0.073	135776
rs2306547	12	26877885	T	C	−0.0211	0.0037	1.79999E-08	0.46	139432
rs7964361	12	1.03E+08	A	G	0.0391	0.0067	4.70002E-09	0.085	139428
rs1351394	12	66351826	C	T	−0.0436	0.0037	1.9002E-32	0.51	143671
rs1819436	13	78580283	C	T	0.0329	0.0057	6.29999E-09	0.87	138979
rs7998537	13	40662742	A	G	−0.0222	0.004	3.89996E-08	0.32	139429
rs7402982	15	99193269	G	A	−0.0232	0.0039	2.30001E-09	0.57	139423
rs12906125	15	91427612	A	G	−0.0228	0.004	0.000000017	0.32	141281
rs144843919	17	29037339	A	G	−0.066	0.0116	1.40001E-08	0.035	121357
rs113086489	17	7171356	T	C	0.0307	0.0038	9.09913E-16	0.56	139426
rs72833480	17	45964861	A	G	0.0226	0.0041	4.60002E-08	0.29	139426
rs753381	20	39797465	C	T	−0.0205	0.0037	2.80001E-08	0.55	143673
rs28530618	20	31275581	G	A	−0.0261	0.0038	7.70016E-12	0.51	138162
rs6016377	20	39172728	T	C	0.0239	0.0039	9.49992E-10	0.43	139425
rs6040076	20	10658882	C	G	0.0231	0.0039	0.000000002	0.49	139424
rs2229742	21	16339172	C	G	−0.036	0.006	2.19999E-09	0.13	143672
rs134594	22	29468456	T	C	−0.0227	0.004	0.00000001	0.65	137340
rs41311445	22	42070374	C	A	−0.0445	0.0066	1.59993E-11	0.098	135729

Abbreviations: EA: effect allele; OA: other allele; EAF: effect allele frequency; *n*: sample size; beta: effect size estimate: SE: standard error; *P*: *p*-value.

Supplementary Table 7. The SNP information of body mass index.

SNP	Chr	Position	EA	OA	Beta	SE	*P*	EAF	*n*
rs543874	1	1.78E+08	G	A	0.0482	0.0039	2.618E-35	0.2667	322008
rs11165643	1	96924097	T	C	0.0218	0.0031	2.07E-12	0.575	320730
rs17024393	1	1.1E+08	C	T	0.0658	0.0088	7.029E-14	0.04167	297874
rs3101336	1	72751185	C	T	0.0334	0.0031	2.661E-26	0.6491	316872
rs2820292	1	2.02E+08	C	A	0.0195	0.0031	1.834E-10	0.5083	321707
rs657452	1	49589847	G	A	−0.0227	0.0031	5.482E-13	0.5833	313651
rs6656785	1	75005776	G	A	0.0217	0.0031	3.829E-12	0.3833	321410
rs1528435	2	1.82E+08	T	C	0.0178	0.0031	1.196E-08	0.5833	321924
rs7599312	2	2.13E+08	A	G	−0.022	0.0034	1.173E-10	0.2917	322024

rs10182181	2	25150296	G	A	0.0307	0.0031	8.776E-24	0.5	321759
rs13021737	2	632348	G	A	0.0601	0.004	1.113E-50	0.875	318287
rs2121279	2	1.43E+08	T	C	0.0245	0.0044	2.313E-08	0.1167	322065
rs12986742	2	58975143	C	T	0.0212	0.0037	1.006E-08	0.5	233833
rs1016287	2	59305625	C	T	−0.0229	0.0034	2.253E-11	0.675	321969
rs2365389	3	61236462	T	C	−0.02	0.0031	1.629E-10	0.3417	316768
rs3849570	3	81792112	A	C	0.0188	0.0034	2.601E-08	0.3667	284339
rs13078960	3	85807590	G	T	0.0297	0.0039	1.737E-14	0.1833	322135
rs16851483	3	1.41E+08	T	G	0.0483	0.0077	3.548E-10	0.0917	233929
rs6804842	3	25106437	G	A	0.0185	0.0031	2.476E-09	0.575	321463
rs1516725	3	1.86E+08	C	T	0.0451	0.0046	1.886E-22	0.9083	320644
rs13107325	4	1.03E+08	T	C	0.0477	0.0068	1.825E-12	0.1167	321461
rs11727676	4	1.46E+08	C	T	−0.0358	0.0064	2.55E-08	0.075	296401
rs10938397	4	45182527	G	A	0.0402	0.0031	3.205E-38	0.4333	320955
rs17001654	4	77129568	G	C	0.0306	0.0053	7.76E-09	0.1583	233722
rs2112347	5	75015242	G	T	−0.0261	0.0031	6.192E-17	0.375	322019
rs13191362	6	1.63E+08	G	A	−0.0277	0.0048	7.339E-09	0.2	321902
rs2033529	6	40348653	G	A	0.019	0.0033	1.388E-08	0.2583	321917
rs9400239	6	1.09E+08	C	T	0.0188	0.0033	1.613E-08	0.7	321988
rs205262	6	34563164	G	A	0.0221	0.0035	1.753E-10	0.2667	315542
rs2207139	6	50845490	G	A	0.0447	0.004	4.126E-29	0.1	322019
rs1167827	7	75163169	G	A	0.0202	0.0033	6.333E-10	0.5417	306238
rs2245368	7	76608143	T	C	−0.0317	0.0057	3.187E-08	0.7583	205675
rs17405819	8	76806584	C	T	−0.0224	0.0033	2.07E-11	0.3667	322085
rs2033732	8	85079709	C	T	0.0192	0.0035	4.889E-08	0.7583	321406
rs10968576	9	28414339	G	A	0.0249	0.0033	6.607E-14	0.2917	322061
rs6477694	9	1.12E+08	T	C	−0.0174	0.0031	2.673E-08	0.6417	322048
rs1928295	9	1.2E+08	C	T	−0.0188	0.0031	7.91E-10	0.425	321979
rs4740619	9	15634326	C	T	−0.0179	0.0031	4.564E-09	0.4667	321887
rs10733682	9	1.29E+08	G	A	−0.0174	0.0031	1.83E-08	0.575	320727
rs7903146	10	1.15E+08	T	C	−0.0234	0.0034	1.112E-11	0.25	322130
rs7899106	10	87410904	G	A	0.0395	0.0071	2.96E-08	0.05	321770
rs17094222	10	1.02E+08	C	T	0.0249	0.0038	5.942E-11	0.2083	321770
rs2176598	11	43864278	C	T	−0.0198	0.0036	2.971E-08	0.8	316848
rs4256980	11	8673939	G	C	0.0209	0.0031	2.9E-11	0.725	320028
rs3817334	11	47650993	T	C	0.0262	0.0031	5.145E-17	0.45	321959
rs12286929	11	1.15E+08	G	A	0.0217	0.0031	1.31E-12	0.4333	321903
rs11030104	11	27684517	G	A	−0.0414	0.0038	5.556E-28	0.2	322103
rs11057405	12	1.23E+08	A	G	−0.0307	0.0055	2.019E-08	0.0917	314111
rs7138803	12	50247468	A	G	0.0315	0.0031	8.153E-24	0.4417	322092
rs12429545	13	54102206	A	G	0.0334	0.0047	1.094E-12	0.1	312934
rs9579083	13	28017270	C	G	0.0295	0.0047	3.461E-10	0.2333	233807
rs10132280	14	25928179	A	C	−0.023	0.0034	1.141E-11	0.3333	321797
rs7141420	14	79899454	T	C	0.0235	0.0031	1.23E-14	0.6167	321970
rs16951275	15	68077168	C	T	−0.0311	0.0037	1.911E-17	0.225	322098
rs3736485	15	51748610	G	A	−0.0176	0.0031	7.412E-09	0.575	321398
rs879620	16	4015729	T	C	0.0244	0.004	1.061E-09	0.5917	233835
rs758747	16	3627358	T	C	0.0225	0.0037	7.473E-10	0.2667	308688
rs9926784	16	19941968	C	T	−0.0265	0.0042	1.849E-10	0.2083	316274
rs3888190	16	28889486	A	C	0.0309	0.0031	3.14E-23	0.3583	321930
rs4889606	16	31011183	G	A	−0.0183	0.0031	4.857E-09	0.3583	321887
rs1558902	16	53803574	A	T	0.0818	0.0031	7.52E-153	0.45	320073
rs12940622	17	78615571	A	G	−0.0182	0.0031	2.494E-09	0.4583	322032
rs1000940	17	5283252	G	A	0.0192	0.0034	1.284E-08	0.225	321836
rs1808579	18	21104888	T	C	−0.0167	0.0031	4.169E-08	0.475	322032
rs6567160	18	57829135	C	T	0.0556	0.0036	3.93E-53	0.2833	321958

rs17066856	18	58049656	C	T	−0.0395	0.0055	6.224E-13	0.1333	319773
rs29941	19	34309532	G	A	0.0182	0.0033	2.407E-08	0.6667	321970
rs17724992	19	18454825	G	A	−0.0194	0.0035	3.415E-08	0.3083	319588
	19	46202172	T	C	−0.036	0.0042	4.585E-18	0.15	300921

Abbreviations: EA: effect allele; OA: other allele; EAF: effect allele frequency; *n*: sample size; beta: effect size estimate: SE: standard error; *P*: *p*-value.

Supplementary Table 8. The SNP information of waist circumference.

SNP	Chr	Position	EA	OA	Beta	SE	*P*	EAF	*n*
rs7550711	1	1.1E+08	T	C	0.058	0.0098	3.40001E-09	0.0339	212149
rs7531118	1	72837239	C	T	0.027	0.0035	1.50003E-14	0.6083	231912
rs3127553	1	49438005	A	G	−0.023	0.0035	1.6E-10	0.6333	231815
rs2820292	1	2.02E+08	C	A	0.019	0.0034	2.39999E-08	0.5083	231899
rs17381664	1	78048331	C	T	0.022	0.0035	4.20001E-10	0.425	232022
rs11165623	1	96893000	A	G	0.02	0.0034	5.19996E-09	0.4833	232057
rs633715	1	1.78E+08	C	T	0.043	0.0043	3.29989E-23	0.2667	218883
rs6545714	2	59307725	A	G	−0.022	0.0035	1.89998E-10	0.625	232046
rs6755502	2	635721	C	T	0.051	0.0045	1.99986E-30	0.875	231849
rs929641	2	58792377	G	A	−0.021	0.0034	1.2E-09	0.3833	231976
rs3849570	3	81792112	A	C	0.021	0.0038	2.19999E-08	0.3667	196103
rs6440003	3	1.41E+08	A	G	0.021	0.0034	2.90001E-10	0.4833	231985
rs2325036	3	85819412	C	A	−0.023	0.0035	2.09991E-11	0.4083	232048
rs1516725	3	1.86E+08	C	T	0.031	0.0051	1.7E-09	0.9083	230610
rs10938397	4	45182527	G	A	0.032	0.0035	6.09958E-20	0.4333	231679
rs2112347	5	75015242	G	T	−0.025	0.0035	3.19963E-13	0.375	232028
rs806794	6	26200677	G	A	−0.022	0.0037	2.1E-09	0.275	225694
rs9400239	6	1.09E+08	C	T	0.024	0.0036	1.9002E-11	0.7	232015
rs2489623	6	1.27E+08	C	A	0.019	0.0034	3.40001E-08	0.5583	231857
rs2033529	6	40348653	G	A	0.021	0.0037	0.000000017	0.2583	232010
rs943005	6	50865820	T	C	0.039	0.0044	7.19946E-19	0.1	232080
rs16894959	6	34825662	C	T	0.026	0.0048	3.40001E-08	0.1	230687
rs10968576	9	28414339	G	A	0.025	0.0036	1.20005E-11	0.2917	232050
rs6163	10	1.05E+08	A	C	0.019	0.0035	3.69999E-08	0.3917	225708
rs7903146	10	1.15E+08	T	C	−0.022	0.0037	3.89996E-09	0.25	232078
rs2293576	11	47434986	A	G	−0.022	0.0036	9.40005E-10	0.3667	226024
rs10840100	11	8669437	G	A	0.02	0.0035	5.39995E-09	0.725	232029
rs10767658	11	27672252	G	C	−0.031	0.0037	3.29989E-17	0.6417	225754
rs7138803	12	50247468	A	G	0.028	0.0035	1.59993E-15	0.4417	232059
rs12429545	13	54102206	A	G	0.031	0.0052	2.5E-09	0.1	222998
rs7144011	14	79940383	T	G	0.033	0.0041	9.3994E-16	0.275	232007
rs10132280	14	25928179	A	C	−0.022	0.0037	2.19999E-09	0.3333	231985
rs4776970	15	68080886	T	A	−0.02	0.0035	2.30001E-08	0.3417	230989
rs749671	16	31088347	A	G	−0.019	0.0035	0.000000032	0.375	232060
rs1558902	16	53803574	A	T	0.074	0.0035	3.6983E-101	0.45	230183
rs2531992	16	4021734	G	A	0.028	0.0048	2.99999E-09	0.8333	232034
rs7498665	16	28883241	G	A	0.034	0.0035	1.39991E-22	0.3583	230193
rs17066856	18	58049656	C	T	−0.037	0.006	8.99995E-10	0.1333	230221
rs6567160	18	57829135	C	T	0.048	0.004	2.60016E-33	0.2833	231894
rs7239883	18	40147671	A	G	−0.021	0.0035	2.30001E-09	0.6833	231731
rs2287019	19	46202172	T	C	−0.035	0.0046	1.69981E-14	0.15	217525
rs16996700	20	50981945	C	T	−0.023	0.0037	1.5E-09	0.3	231903

Abbreviations: EA: effect allele; OA: other allele; EAF: effect allele frequency; *n*: sample size; beta: effect size estimate: SE: standard error; P: *p*-value.

Supplementary Table 9. Details of the 49 items used to compose the Frailty Index [43].

Type of deficit	Item	Trait	Categories	Coding in FI item	Score of 1 *n* (%)**
Sensory	1	Glaucoma*	no, yes	Categorised 0/1	3,728 (2.26)
	2	Cataracts*	no, yes	Categorised 0/1	8,993 (5.46)
	3	Hearing difficulty	no, yes, completely deaf	Categorised 0/1 (combined yes/deaf groups as 1)	52,506 (31.90)
Cranial	4	Migraine*	no, yes	Categorised 0/1	3,863 (2.35)
	5	Dental problems	ulcers, painful gums, bleeding gums, loose teeth, toothache, dentures	Categorised 0/1 for none vs. any	71,494 (43.43)
Mental wellbeing	6	Self-rated health	excellent, good, fair, poor	0 – excellent; 0.25 – good; 0.5 - fair; 1 – poor	6,231 (3.79)
	7	Fatigue: frequency of tiredness/lethargy in last two weeks	not at all, several days, more than half, nearly every day	0, 0.25, 0.5, 1, respectively	6,928 (4.21)
	8	Sleep: experience of sleeplessness/insomnia	never/rarely, sometimes, usually	Categorised 0, 0.5, 1, respectively	49,304 (29.95)
	9	Depressed feelings: frequency in last two weeks	not at all, several days, more than half, nearly every day	0 – not at all, 0.5 – several days, 0.75 - more than half, 1 – nearly every day	1,834 (1.11)
	10	Self-described nervous personality	no, yes	Categorised 0/1	35,478 (21.55)
	11	Severe anxiety/panic attacks*	no, yes	Categorised 0/1	1,936 (1.18)
	12	Common to feel loneliness	no, yes	Categorised 0/1	24,005 (14.58)
	13	Sense of misery (ever/never)	no, yes	Categorised 0/1	57,738 (35.08)
Infirmity	14	Infirmity: long-standing illness or disability	no, yes	Categorised 0/1	60,831 (36.95)
	15	Falls in last year	categorical: no falls, one fall, more than one	0, 0.5, 1, respectively	10,831 (6.58)
	16	Fractures/broken bones in last five years	no, yes	Categorised 0/1	15,881 (9.65)
Cardiometabolic	17	Diabetes*	no, yes	Categorised 0/1	10,881 (6.61)
	18	Myocardial infarction*	no, yes	Categorised 0/1	6,221 (3.78)
	19	Angina*	no, yes	Categorised 0/1	8,779 (5.33)
	20	Stroke*	no, yes	Categorised 0/1	3,756 (2.28)
	21	High blood pressure*	no, yes	Categorised 0/1	59,258 (36.00)
	22	Hypothyroidism*	no, yes	Categorised 0/1	9,586 (5.82)
	23	Deep-vein thrombosis*	no, yes	Categorised 0/1	4,450 (2.70)
	24	High cholesterol*	no, yes	Categorised 0/1	45,052 (27.37)
Respiratory	25	Breathing: wheeze in last year	no, yes	Categorised 0/1	34,566 (21.00)
	26	Pneumonia*	no, yes	Categorised 0/1	2,800 (1.70)
	27	Chronic bronchitis/emphysema*	no, yes	Categorised 0/1	3,839 (2.33)
	28	Asthma*	no, yes	Categorised 0/1	17,447 (10.60)
Musculoskeletal	29	Rheumatoid arthritis*	no, yes	Categorised 0/1	2,314 (1.41)
	30	Osteoarthritis*	no, yes	Categorised 0/1	19,804 (12.03)
	31	Gout*	no, yes	Categorised 0/1	3,208 (1.95)
	32	Osteoporosis*	no, yes	Categorised 0/1	4,187 (2.54)
Immunological	33	Hayfever, allergic rhinitis or eczema*	no, yes	Categorised 0/1	32,727 (19.88)
	34	Psoriasis*	no, yes	Categorised 0/1	1,763 (1.07)
Cancer	35	Any cancer diagnosis*	no, yes	Categorised 0/1	19,068 (11.58)
	36	Multiple cancers diagnosed (number reported)	Range from 0 to 6	0 - no cancer or single cancer, 1 - multiple cancers	1,430 (0.87)
Pain	37	Chest pain	no, yes	Categorised 0/1	25,041 (15.21)
	38	Head and/or neck pain	no, yes (combining responses to pain in head and neck/shoulders)	Categorised 0/1	49,029 (29.78)

	39	Back pain	no, yes	Categorised 0/1	40,764 (24.76)
	40	Stomach/abdominal pain	no, yes	Categorised 0/1	10,703 (6.50)
	41	Hip pain	no, yes	Categorised 0/1	21,192 (12.87)
	42	Knee pain	no, yes	Categorised 0/1	37,661 (22.88)
	43	Whole-body pain	no, yes	Categorised 0/1	2,468 (1.50)
	44	Facial pain	no, yes	Categorised 0/1	2,402 (1.46)
	45	Sciatica*	no, yes	Categorised 0/1	1,643 (1.00)
Gastrointestinal	46	Gastric reflux*	no, yes	Categorised 0/1	8,371 (5.09)
	47	Hiatus hernia*	no, yes	Categorised 0/1	5,076 (3.08)
	48	Gall stones*	no, yes	Categorised 0/1	3,593 (2.18)
	49	Diverticulitis*	no, yes	Categorised 0/1	2,748 (1.67)

*Participants reported medically diagnosed conditions for these items. ***N* = 164,610 (60–70 year olds; European descent; complete case analysis of all 49 FI components).
